# Acupoint specificity on acupuncture regulation of hypothalamic- pituitary-adrenal cortex axis function

**DOI:** 10.1186/s12906-015-0625-4

**Published:** 2015-03-27

**Authors:** Shao-jun Wang, Jiao-jiao Zhang, Hao-yan Yang, Fang Wang, Si-ting Li

**Affiliations:** China Academy of Chinese Medical Sciences, Institute of Acupuncture and Moxibustion, Dong Zhi Men Nei Nan Xiao Street, No.16, Beijing, 100700 China; Chinese Academy of Medical Sciences (CAMS) &Comparative Medicine Centre, Institute of Laboratory Animal Scineces, Peking Union Medical College (PUMC), Pan Jia Yuan Nan Li No.5, Chao Yang District, Beijing, 100021 China

**Keywords:** Acupuncture, Stress reaction neurons, Hypothalamic paraventricular nucleus, Hypothalamic-pituitary-adrenal cortex axis, Unpredictable chronic mild stress

## Abstract

**Background:**

The hypothalamus is an essential part of the brain that responds to a variety of signaling including stressful stimulations and acupuncture signals. It is also the key element of the hypothalamic-pituitary-adrenal cortex axis(HPAA). The effect of acupuncture is transmitted into the brain from the distance sensory receptor around the acupoints via peripheral nerves and body fluid. In vivo recording the activities of stress reaction neurons (SRNs, CRH-like neurons) in hypothalamic paraventricular nucleus (PVN) in response to the stimulations from different acupoints could therefore objectively reflect the acupuncture afferent effect.

**Methods:**

In this study, the electrophysiological method was adopted to record synchronously the activities of 43 CRH-like neurons after acupuncture stimulations at 33 acupoints located at the different regions. The acupoints that specifically activate certain CRH-like neurons (specificity acupoints) were selected. Furthermore, we investigated in a rat model of unpredictable chronic mild stress (UCMS) whether these specificity acupoints regulate HPAA function. The endpoints of measurement include corticosterone (CORT) level in peripheral blood, the expressions of corticotrophin releasing hormone (CRH) and glucocorticoid receptor (GR) protein in PVN and the animal behavioral performance.

**Results:**

Our results reveal that Shenshu (BL23), Ganshu (BL18), Qimen (LR14), Jingmen (GB25), Riyue (GB24), Zangmen (LR13), Dazui (DU14) and auricular concha region (ACR) are the specificity acupoints; and Gallbladder, Liver and Du Channels were the specificity Channels. The acupoints on Gallbladder Channel and the acupoints innervated by the same spinal cord segments as the adrenal gland demonstrated dramatic effects.

**Conclusions:**

This study provides a new platform to further explore acupoints specificity in the regulation of HPAA activities.

## Background

Hypothalamic paraventricular nucleus (PVN) contains the main nuclei that secrete stress hormones. Particularlly, the paravicellular subnucleus of PVN contains numerous neurons roducing corticotripin releasing hormone (CRH), enkephalin, or thyrotropin releasing hormone (TRH). These hormones are the principal components of stress responses through hypothalamus-hypophysis axis [1]. When the stressor arrives at PVN neurons, CRH neurons synthesize and release CRH to the pituitary gland, and stimulate the pituitary gland to secrete adrenocorticotropic hormone (ACTH). ACTH enters blood circulation and acts on the adrenal gland to promote the synthesis and secretion of glucocorticoids. The exogenous glucocorticoids regulate the secretion of CRH from PVN via feedback regulation on hypothalamic-pituitary- adrenal cortex axis (HPAA), reduce stress response, influence responses in hippocampus and other limbic system, and impact the arousal, cognition and affection [2,3]. The negative feedback regulation of glucocorticoid on HPAA is achieved via acting on the GR and mineralocorticoid receptor (MR) that are widely distributed in the central nervous system [4].

Some study showed that the expression of estrogen receptor (ER) and androgen receptor (AR) increased in the CRH neurons of PVN in depression patients. The sexual hormone binds to the sexual hormone receptors on CRH neurons and directly regulate the activity of CRH neurons [5,6]. The estrogen increases the expression of CRH, while the androgen inhibits the activity of CRH [7,8].

Although it has been proven that acupuncture could regulate the function of HPAA [9-11], the acupoint specificity in the acupuncture-afforded regulation on HPAA is not clear, resulting in the lack of guideline for acupoint selection in clinical practice and limited clinical efficacy.

Our group had applied the in vivo electrophysiology method to record activities of CNS neurons in our previous studies [12,13]. In this study, the noxious heat stimulus (a stress stimulus) and hydrocortisone (a corticosteroid) sensitive neurons in PVN were recorded. The neurons that involved in stress reaction were further determined and selected via their reaction to sexual hormones. By taking neuronal discharge as an index, we observed the differences in the neuronal activities elicited by acupuncture at 33 acupoints of different regions, and chose the acupoints that specifically activate a certain neuron (specificity acupoints). We further analyzed the correlation of the specificity acupoints with the related Channels and the dominant spinal cord segments. Additionally, through the effects of the specificity acupoints on the CORT level in peripheral blood, and on the expressions of CRH and GR protein in PVN in unpredictable chronic mild stress (UCMS) model rats, the effects of the acupoints were verified so as to further explore the acupoint specificity on the regulation of PHAA function. This study will provide theoretical evidences of acupoint selection in clinical practice of acupuncture to regulate homeostasis and enhance the adaption to external environment.

## Methods

### Animals

Male Sprague-Dawley rats in 150–170 g were obtained from the Laboratory Animal Resources Center, National Institute for the Control of Pharmaceutical and Biological Products, Beijing (Certificate No. SCXK (jing) 2009-0017). These animals were individually caged on a 12 h light/dark cycle (lights on at 8:00 a.m., lights off at 8:00 p.m.) under controlled temperature (22 ± 1°C) and humidity (50% ±5%) conditions. Standard rat chow and water were given ad libitum. Animals were allowed to acclimatize for seven days before the study. All experiment procedures comply with the guidelines of the “Principles of Laboratory Animal Care” (NIH publication number 80-23, revised 1996) and the legislation of the People’s Republic of China for the use and care of laboratory animals. The experimental protocols were approved by the Animal Experimentation Ethics Committee of the Institute of Acupuncture and Moxibustion, China Academy of Chinese Medical Sciences. Efforts were made to minimize the number of animal use and the suffering of the experimental animals.

### Modeling

Rats were initially anesthetized with an intraperitoneal injection of urethane (1.0 –1.2 g/kg; Duxin-Fine Preparation Factor, Beijing, China). Rats were tracheotomized to prevent from the aspiration of saliva and, when required, to perform artificial ventilation. Catheters were inserted into the left carotid artery and the jugular vein for blood pressure monitoring and drug injection, respectively. Heart rate and blood pressure were continuously monitored and body temperature was maintained around 37°C by means of a feedback- controlled homeothermic blanket.

The animal was placed in a stereotaxic head frame (KOPF-900; Tokyo, Japan), the skull was fixed and leveled between bregma and lambda. A craniotomy was performed to remove bone overlying the cortex, and this allowed the glass microelectrode to be lowered into the hypothalamus and covered with liquid paraffin. The locations of the PVN region was defined based on the brain atlas of Paxinos and Watson [14]. The three-dimensional coordinates of PVN: 0.4–0.9 mm lateral to and 6.9–7.4 mm anterior to the lambda, 7.0–8.0 mm in depth. The hole was drilled on the skull in the center of the scope at first, and then, the cranial parietal bone was removed carefully with rongeur along the drilled hole and finally, the meninges was removed with gossamer under the microscope.

### Records of the spikes of SRN in PVN and the activation effect of the different acupoints

Spontaneous discharges from individual neurons encountered within PVN were recorded extracellularly using glass microelectrodes filled with a solution of 1.0 M NaCl and pontamine sky blue (tip impedance 15–20 MΩ). The isolated action potentials were fed into a window discriminator and displayed on an oscilloscope screen (VC-10, Nihon Kohden, Tokyo, Japan). The outputs of the window discriminator and amplifier were fed into a data collection system and a personal computer data acquisition system (Power Lab) to compile peristimulus time histograms or wavemark files for further analysis. The spontaneous firing rate of PVN neurons was continuously monitored for 5 to 10 minutes before and after stimulation. As there was the often moment-to-moment fluctuation in PVN neuronal excitability that was associated with a change in background activity, the impulse counts in association with a stimulation procedure were compared with the background activity for the 30-second period immediately before the stimulation took place (“net response”).

After recording the spontaneous discharges in PVN, the operations were done as follows: The posterior 2/3 of the rat tail was soaked in 48°C hot water. If the neuronal spikes were enhanced or reduced (by ≥ 20%), this neuron would be indicated as PVN stress sensitive neuron. At this moment, the intravenous injection of hydrocortisone (Sigma-H0888, the concentrated solution was prepared with anhydrous ethanol, and diluted with 0.9% physiological saline, 10 mg/kg for intravenous injection) [15] was followed. If the spikes were inhibited (by ≥ 20%), testosterone 8 mg/kg (Sigma -T1500, 30 ~ 50 ng/kg) and β-estradiol (Sigma -E8875, 30–50 ng/kg) [12,13] would be injected separately when the spikes were recovered near to the spontaneous discharges. Regarding to the enhancement or inhibition of stress stimulation, the SRN involved in PVN would be determined if inhibited obviously with the intravenous injection of hydrocortisone, slightly inhibited with testosterone and weakly responded to estrogen separately.

Taking the neuron discharge as the index, the observation was performed at 33 acupoints of the different regions with acupuncture, namely Ganshu (BL18), Shenshu (BL23), Chengshan (BL57), and Dashu (BL11) of the Bladder Channel; Jingmen (GB25), Yanglingquan (GB34), Riyue (GB24) and Fengchi (GB20) of the Gallbladder Channel; Tianshu (ST25), Zusanli (ST36), Wuyi (ST15) and Futu (ST32) of the Stomach Channel; Zhubin (KI9), Shangqu (KI17) and Futonggu (KI20) of the Kidney Channel; Taichong (LR3), Zhangmen (LR13) and Qimen (LR14) of the Liver Channel; Sanyinjiao (SP6), Fuai (SP16) and Daheng (SP15) of the Spleen Channel; Hegu (LI4) of the Large Intestine Channel, Waiguan (TE5) of the Triple Energizers Channel, Yanglao (SI6) of the Small Intestine Channel, Taiyuan (LU9) of the Lung Channel, Neiguan (PC6) of the Pericardium Channel, Shenmen (HT7) of the Heart Channel, Shuigou (DU26), Dazhui (DU14) and Mingmen (DU4) of Du Channel, Shanzhong (RN17) and Zhongwan (RN14) of Ren Channel and Auricular Concha Region (ACR). The differences in the activation effect and intensity of neurons were observed, and the specificity acupoints for activating these neurons with acupuncture were selected. And the correlation of this specificity acupoints with the related Channels and the dominant spinal nerve segments was analyzed. There was a certain interval of stimulation among the different acupoints. After the stimulation at the previous acupoint, the next acupoint was followed when the neuron spikes were recovered to be the spontaneous discharge.

To verify whether the recorded sites were in PVN or not, the microelectrode electrophoresis technology was adopted at the end of record, in which pontamine sky blue infusion were used for the localization [12]. Under anesthesia by diethyl ether in the animal, the heart perfusion technique was applied with 4% formalin. The brain tissue was collected and prepared as frozen slices (20 μm) after 10% and 20% sucrose gradient dehydration. The labeled sites were observed under microscope. The neuron unit recorded definitely in PVN would be included in the statistical analysis.

### Behavior test

#### Open Field Test

The open field apparatus was constructed of black plywood and measured 80 × 80 cm with 40 cm walls. White lines were drawn on the floor. The lines divided the floor into twenty-five 16 × 16 cm squares. A central square (16 cm × 16 cm) was drawn in the middle of the open field. Rats were put on the central square, at the same time the video camera was turned on for video recording from the top of the open field apparatus. Behaviors of rats were recorded for 3 minutes, with the grid number being counted as the horizontal score and the time of both frontal claws uplifting from the ground as the vertical score, with the 10 cm lines long being counted as travel along the wires score [16,17]. Only one animal was used in one determination. Each determination lasted 3 min. The next determination with another rat began after thoroughly cleaning the box. Each animal was determined before and after modeling, as well as after acupuncture separately.

#### Sucrose consumption

The 24 h water fasting was required in all the rats before and after modeling, as well as after acupuncture (after open-field test), but 1% sucrose water was allowed freely for 24 h. The sucrose consumption was observed in 24 h for the animals of each group.

### Establishment of unpredictable chronic mild stress (UCMS) model

Fifty-eight of one hundred rats were recruited with the total score of 60 ~ 120 in the open field test [16] and evenly randomized into 5 groups. A successful UCMS model rat was created with the score of the open field test equal or minus 60. Qualified rats were distributed into 5 groups: a control group (N group, no acupuncture, n = 10), a target -acupoints group (NEA group, Qimen (LR14) and Shenshu (BL23), n = 12), an UCMS group (M group, no acupuncture, n = 12), an UCMS non-target- acupoints group (MNEA group, Sanyinjiao (SP6) and Zhubin (KI9), n = 12), and an UCMS target-acupoints group (MEA group, Qimen(LR14) and Shenshu (BL23), n = 12). Every five rats in the N and NEA group were housed in one cage. However, rats in the M, MEA, and MNEA groups were caged individually. Depression model was established by 21 days of UCMS combined with isolation. UCMS procedures were based on published studies [17,18], including 7 kinds of stressors: food deprivation, water deprivation, cage tilt 45°C (Ugo Basile s.r.l. hot/cold plate, Model 35100–001, Italy), swimming in 4°C water, clipping tail 3 min, 50 V electric shock (Electronic stimulator, NIHON KOHDEN, Japan) and overnight illumination. The stressors were given randomly 3 times daily for 21 continuous days. The rats in the N and NEA group were housed without any external stimuli except for necessary procedures such as routine cage cleaning.

### Western Blot (WB) analysis

Western blot analysis was performed as followed [19]: Rats tissues of brain (PVN area), was homogenized on ice in RIPA buffer (50 mol/L Tris–Cl, pH 7.6, 5 mol/L ethylenediaminetetraacetic acid, 150 mol/L NaCl, 0.5% Nonidet P-40, 0.5% Triton X-100) containing protease inhibitor cocktail and phosphatase inhibitor cocktails I/II (Sigma-Aldrich). The homogenate was centrifuged at 12, 000 xg for 30 minutes at 4°C. The supernatant were collected and the protein concentration was measured using the Bradford assay. Twenty micrograms of the sample was separated with 10% polyacrylamide gel blotted on a PVDF film (Millipore Corp). The blotted film was blocked for 2 hours at 4°C in blocking solution (1 × TBS with 5% non-fat milk and 0.02% Tween 20). The blocked film was shaken overnight at 4°C using primary antibodies in blocking solution. Following three times washes with TBST (1 × TBS with 0.02% Tween 20), the film was shaken for 1 hour at room temperature with peroxidase-conjugated secondary antibody, and then washed three times with TBST. Detection was performed using an ECL kit (Thermo #OC183596) according to the manufacturer’s instructions. The western blots shown are representative of at least three independent experiments.

The antibodies used included the following: anti-CRH (Datasheet, #10944-1-AP) (1:500), anti-GR(Santa Cruz, #H-300) (1:1000), anti-β-Actin (Sigma, A5316) (1:10000), HRP-conjugated IgG secondary antibodies (1:2000) (GE Healthcare Life Sciences). All western blot data were analyzed by Image J software.

### Enzyme linked immunosorbent assay (ELISA) tests

Corticosterone was determined using an ELISA assay (ELISA Kit, Assay pro, USA, #EC3001-1). All ofthe reagents and samples were prepared at room temperature before use and the samples were centrifuged again after thawing before the assay. The method of test was in compliance with the procedure of kit.

### Experimental procedures

The open field test was conducted on the -1th, 23th and 29th days of the study in the rats. The sucrose consumption test was on the 0th, 24th and 30th days. UCMS model was determined on the 24th day. At the end of modeling, acupuncture was given continuously for 6 days, once every day, 30 min each time. Acupuncture was applied to bilateral BL23 and LR14 in MEA group, to SP6 and KI9 in MNEA group and to BL23 and LR14 in NEA group. Acupuncture was not applied in N and M groups. The parameters of electro-acupuncture (EA): 2Hz, 2.0 mA, 30 min. Acupuncture was performed under the inhaled anesthesia with isoflurane. On the 30th day at the end of acupuncture, the rats were sacrificed immediately after the last sucrose consumption test. ELISA was used to detect the concentration of plasma corticosterone. WB method was adopted to detect the expressions of CRH and GR protein in PVN. As a result, it was to explore whether the acupoints for the specific activation on stress reaction and hydrocortisone sensitive neurons were also the specificity acupoints for the regulation of corticosterone secretion as well as the expressions of CRH and GR protein in PVN. The same inhaled anesthesia was also applied in N group and NEA group.

### Statistical analysis

The statistical analysis was performed by using one-way analysis of variance (ANOVA) following by a Turkey test with software SPSS 13.0. P < 0.05 was considered statistically significant, and the data were expressed as means ± SEM.

## Results

### General features of PVN neurons

**Figure 1:**
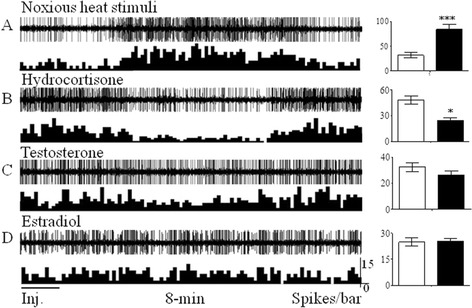
**Extracellular electrophysiological records from the excitatory CRH-like neurons in PVN (after noxious heat stimulus or Inj., 8-min).** The left figures showed the representative neuronal activities in PVN under the different stimulus. The statistical analysis of the spikes of the corresponding neurons was illustrated on the right column, in which, the white histograms recorded the spontaneous discharges of the neurons at the unit time in PVN, and the black histograms illustrated the firing of neurons at the unit time after stimulation. The neuron firing was increased suddenly by noxious heat stimulation **(A)**, inhibited by hydrocortisone **(B)**, partially suppressed by testosterone **(C)** and slightly responded by estrogen **(D)**. *p < 0.05, ***p < 0.001 *vs*. spontaneous discharges of the neurons.

In this study, the spikes of 653 neurons in PVN were recorded in 172 male SD rats. 163 neurons responded to hot water stimulus at 48°C (noxious stimulates), (24.96% of the total neurons recorded); of which, 121 neurons exhibited the intensive reaction (Figure 1A, the neuron firing was in the range from 34.13 ± 4.27 to 89.08 ± 14.65 spikes/second, and the excitatory rate was 161%) and 42 neurons presented the inhibitory reaction. The rest 490 neurons had no response to hot water stimulus at 48°C (accounting to 75.04% of the total neurons recorded).

We randomly selected 10 non-responded neurons. After determining that these neurons had no reaction to the noxious stimulus, we increased the stimulating temperature from 48°Cup to 58°C and observed the changes of neurons in PVN. Of those 10 neurons, the firing of 1 neuron was slightly stronger after temperature increase while the firing of the rest 9 neurons did not change, indicating that these 490 neurons belong to the non-stress reaction-related neurons in PVN.

### Feedback inhibitory effect of reactive neurons in PVN

In order to further determine whether or not the recorded neurons in response to noxious stimulus were related to stress reaction, we selected 47 neurons from 121 excitatory neurons and injected hydrocortisone intravenously. We found that the activity was inhibited obviously in 43 neurons and the neuron firing reduced from 47.42 ± 8.71 to 24.16 ± 5.59 spikes/second, and the inhibitory rate was 42.54% (Figure 1B). We selected 13 neurons from these 43 excitatory neurons for comparison. When the firing reduced and was recovered to be normal after injection of hydrocortisone, we injected testosterone and estrogen separately in those 13 neurons It was demonstrated that the firing of 13 neurons was inhibited slightly after injection of testosterone (Figure 1C, from 31.19 ± 5.65 to 26.73 ± 6.22 spikes/second, and the inhibitory rate was 14.30%), and, it increased slightly after the injection of estrogen (Figure 1D, increased from 24.25 ± 4.25 to 26.32 ± 3.21 spikes/second and the excitatory rate was 8.54%).

**Figure 2:**
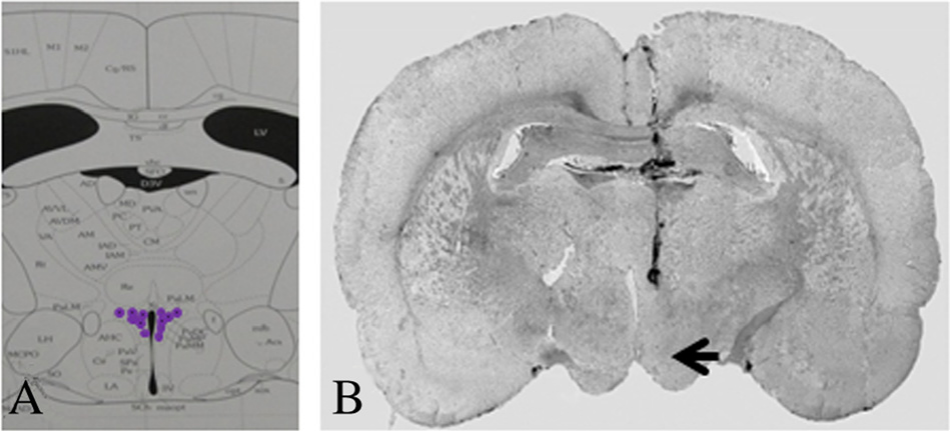
**The site of recorded neurons in PVN** ***.*** **A** showed the concept map in PVN (blue marker positions). **B** showed the recording site with pontamine sky blue labeled in the PVN (Arrow marker).

The results showed that those 43 excitatory neurons exhibited obviously excitatory reaction to noxious heat stimulus and to the inhibition to hydrocortisone. Their reactions, however, were not obvious to testosterone and estrogen, suggesting that these neurons were CRH-like neurons in PVN. Figure 2B illustrated the sites of stress reactive neurons recorded in the experiment.

### Activation effect of acupuncture at different acupoints on the excitatory CRH-like neurons in PVN

**Figure 3:**
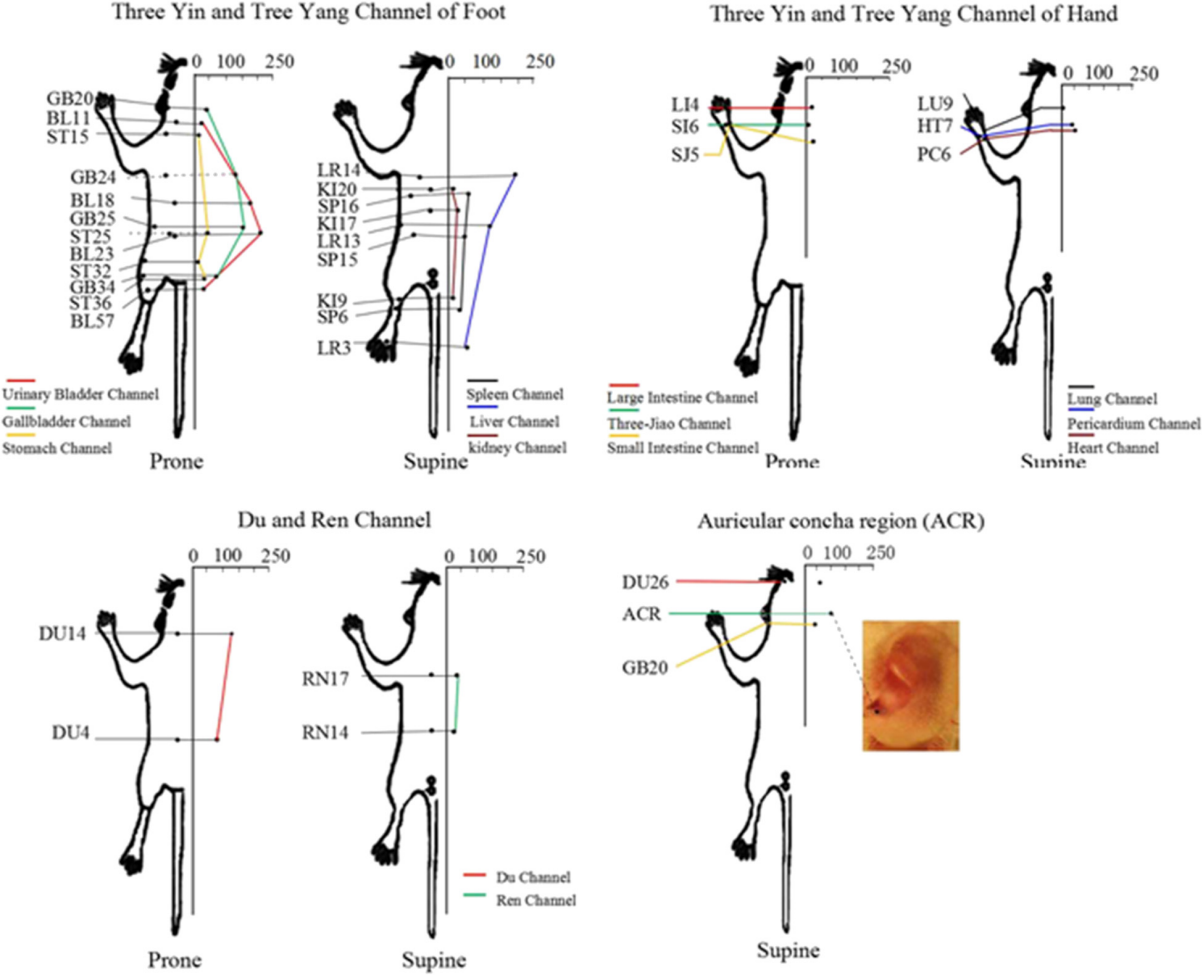
**Demonstrated the activation effect of acupuncture at 33 acupoints of different regions on the excitatory CRH-like neurons in PVN among 33 acupoints.** The abscissa represented the increasing percentage of the excitatory CRH-like neurons after different acupoints stimulation compared with the spontaneous discharges The acupoints of three Yin (Supine) and three Yang (Prone) Channels of Foot showed at upper left, the acupoints of three Yin (Supine) and three Yang (Prone) Channels of Hand showed at upper right, the acupoints of Du (Prone) Channel and Ren (Supine) Channel showed at lower left, the acupoints of ACR, hand and face showed at lower right.

The experiment demonstrated the activation effect of acupuncture at 33 acupoints of different regions on the excitatory CRH-like neurons in PVN. The effect was compared among the acupoints at the different dominant spinal cord segments of the same Channel, those at the same or adjacent dominant spinal cord segments, as well as those at the different Channels (Figure 3).

The acupoints with activation effect were determined if the spikes were increased by ≥20% after acupuncture as compared with the spontaneous discharge. Of 33 acupoints observed in this study, 24 acupoints presented the activation effect. The sequence was: Shenshu (BL23), Qimen (BL14), Ganshu (BL18) > Jingmen (GB25), Dazhui (DU14) > Riyue (GB24), Zhangmen (LR13) > ACR > Yanglingquan (GB34), Fuai (SP16), Shuigou (DU26), Mingmen (DU4), Taichong (LR3), Neiguan (PC6), Shanzhong (RN17), Tianshu (ST25), Fengchi (GB20), Zusanli (ST36), Sanyinjiao (SP6) > Shenmen (HT7), Daheng (SP15), Shangqu (KI17), Waiguan (SJ5), Zhongwan (RN14).

In comparison among the acupoints of the different Channels, located on the same or adjacent spinal nerve segments as the adrenal gland, there was significant difference in comparison of Shenshu (BL23) and Qimen (LR14) with Riyue (GB24) and Zhangmen (LR13) (*p* < 0.05). The difference was extremely significant in comparison of Shenshu (BL23), Qimen (LR14), Ganshu (BL18), Jingmen (GB25) and Dazhui (DU14) with Tianshu (ST25, Daheng (SP15), Fuai (SP16), Shangqu (KI17), Futonggu (KI20), Zhongwan (DU14) and Mingmen (DU4) (*p* < 0.01). The difference was significant in comparison of Riyue (GB24) and Zhangmen (LR13) with Tianshu (ST25), Shangqu (KI17) and Futonggu (KI20) (*p* < 0.05).

In comparison of the acupoints on the trunk, head and face at the different spinal nerve segments as the adrenal gland, the experiment observed the effects on 7 acupoints, named ACR, Dazhui (DU14), Shuigou (DU26), Fengchi (GB20), Wuyi (ST15), Dashu (BL11) and Shanzhong (RN17). The results showed that the difference was significant in comparison of Shenshu (BL23), Qimen (LR14) and Ganshu (BL18) with ACR, Shuigou (DU26), Fengchi (GB20), Wuyi (ST15), Dashu (BL11) and Shanzhong (RN17) (*p* < 0.05), and the differences were significant in comparison of Dashu (BL11) and ACR with Fengchi (GB20), Wuyi (ST15), Dashu (BL11) and Shanzhong (RN17).

In comparison of the acupoints of the different Channels at the hindlimb, but at the same dominant spinal nerve segments, the activation effects were different among Futu (ST32), Zusanli (ST36), Sanyinjiao (SP6), Yanglingquan (GB34), Zhubin (KI9), Chengshan (BL57) and Taichong (LR3), but without statistical significance indicated. The difference was extremely significant as compared with Shenshu (BL23), Qimen (LR14), Ganshu (BL18), Jingmen (GB25) and Dazhui (DU14), and was significant as compared with Riyue (GB24) and Zhangmen (LR13). The >statistical results for the acupoints at the forelimb, named Neiguan (PC6), Shenmen (HT7), Taiyuan (LU9), Hegu (LI4), Waiguan (SJ5) and Yanglao (SI6) were same as those acupoints at the hindlimb.

In comparison among the acupoints at the same Channel, but at different dominant spinal nerve segments, the differences were significant in comparison of Ganshu (BL18) and Shenshu (BL23) with Chengshan (BL57) and Dazhu (BL11), in comparison of Jingmen (GB25) and Riyue (GB23) with Yanglingquan (GB34) and Fengchi (GB20), in comparison of Qimen (BL14) and Zhangmen (LR13) with Taicong (LR3) and in comparison of Dazhui (DU14) with Mingmen (DU4) and Shuigou (DU26).

In summary, the acupoints of Liver and Gallbladder Channels and Du Channel at the different dominant spinal nerve segments all presented the activation effects, especially, the effect was more obvious for those acupoints located at the same or adjacent dominant spinal nerve segments as the adrenal gland. The response was quite obvious at the dominant segments of Bladder Channel, but the distal acupoints presented weaker activation effect. Even though the activation effects all indicated at the acupoints of Stomach, Spleen, the Triple Energizer, Heart and Pericardium Channels, the responses were weaker as compared with Liver, Gallbladder, Du and Bladder Channels. The activation effect of the acupoints of Du Channel was more obvious than that of those acupoints of Ren Channel, located at the same dominant spinal nerve segments. It was necessary to emphasize that the activation effect of ACR was quite obvious particularly.

### Effects of acupuncture at different acupoints on behaviors in UCMS model rats

The animal activity in five groups was gradually decreasing after modeling and after treatment as compare with that before modeling. Table 1 showed the normalized data [19] for the animal movement in three directions of the open field test and sucrose consumption in the five groups (after modeling and after treatment). The results showed: the horizontal, vertical and along-the-line movements in M, MNEA and MEA groups were significantly decreased as compared with N and NEA groups in the open field test after modeling (*p* < 0.05, 0.01, 0.001), the sucrose consumption was also decreased remarkably (*p* < 0.05). After acupuncture treatment, the difference in the horizontal movement was the most obvious among the five groups. The decrease degree of horizontal movement in M group was the most remarkable, followed by MNEA, MEA, N and NEA groups in succession. The differences were extremely significant in M and MNEA groups as compared with N and NEA groups(*p* < 0.01), the differences were significant in MEA group as compared with N and NEA groups(*p* < 0.05), the differences were significant in MEA group as compared with M group(*p* < 0.05). The differences in the vertical movements were not significant in comparison among the groups. The differences were extremely significant in M, MNEA and MEA groups as compare with N and NEA groups (*p* < 0.001) in along-the-line movement, but there was no improvement after treatment. Concerning to sucrose consumption analysis, it was significantly reduced in M group, indicating the extremely significant differences as compared with N and NEA groups (*p* < 0.001). The difference was significant in MNEA group as compared with N and NEA groups (*p* < 0.05). The difference was not significant in MEA group as compared with N and NEA groups. The differences were significant between MNEA group and M group (*p* < 0.05), as well as between MEA group and MNEA group.

**Table 1:** **The effects of different acupoints on the open field test and sucrose consumption of rats**

**Behavior test**	**Normalized data**	**Day**	**N group**	**NEA group**	**M group**	**MNEA group**	**MEA group**
Open Field Test	Horizontal movement	23th	0.76 ± 0.17	0.70 ± 0.17	0.23 ± 0.07***	0.17 ± 0.05***	0.24 ± 0.09***
		29th	0.40 ± 0.10	0.44 ± 0.05	0.03 ± 0.01**	0.06 ± 0.02**	0.20 ± 0.03*#
	Vertical movement	23th	0.64 ± 0.04	0.65 ± 0.22	0.33 ± 0.08*	0.22 ± 0.08*	0.23 ± 0.06*
		29th	0.27 ± 0.09	0.35 ± 0.15	0.12 ± 0.02	0.21 ± 0.05	0.21 ± 0.05
	Along-the-line movement	23th	0.80 ± 0.03	0.76 ± 0.08	0.34 ± 0.13**	0.12 ± 0.08**	0.23 ± 0.08**
		29th	0.56 ± 0.03	0.58 ± 0.08	0.14 ± 0.08***	0.12 ± 0.05***	0.10 ± 0.06***
Sucrose consumption		24th	1.83 ± 0.17	1.90 ± 0.23	1.23 ± 0.20*	1.20 ± 0.15*	1.17 ± 0.15*
		30th	2.17 ± 0.13	2.44 ± 0.26	1.31 ± 0.13***	1.79 ± 0.10*#	2.30 ± 0.06###△

The data showed that the normalized data of behavior test score after modeling and after treatment. M was UCMS group, MEA was UCMS target acupoints group, MNEA was UCMS non-target acupoints group, N was control group, NEA was control target acupoints group. The data were expressed as mean ± SEM in each group. ^*^ *p* < 0.05, ^**^ *p* < 0.01, ^***^ *p* < 0.001, *vs*. N group. #*p* < 0.05, ###*p* < 0.001 *vs*. M group. △*p* < 0.05, MNEA *vs*. MEA group. Normalized data = tested value on the 23rd, 24th, 29th or 30th day / tested value on -1th day or 0^th^ day in same animal separately [19].

### Effect of acupuncture at the different acupoints on plasma CORT level

**Figure 4:**
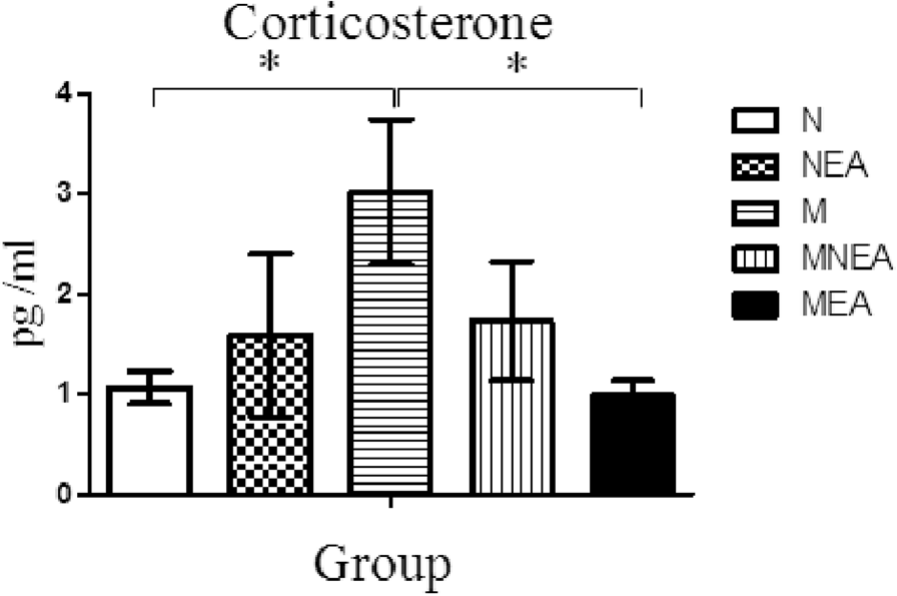
**Effect of acupuncture at the different acupoints on plasma CORT level.** The results showed that the CORT level in M group was significantly higher than that in N and MEA group (*p < 0.05).

Experiments compared the effects on CORT levels between MEA group and MNEA group in UCMS model rats (Figure 4). The results showed: the CORT level of peripheral blood in M group was significantly higher than that in N group (*p* <0.05). The level was significantly inhibited in MEA group, indicating a significant difference as compared with M group (*p* < 0.05). The level was also reduced in tendency in MNEA group, but without significant difference indicated as compared with M group (*p* > 0.05). The level was increased in NEA group, but there was no significant difference as compared with N group (*p* > 0.05).

### Effect of acupuncture at the different acupoints on the expressions of GR and CRH protein in PVN

**Figure 5:**
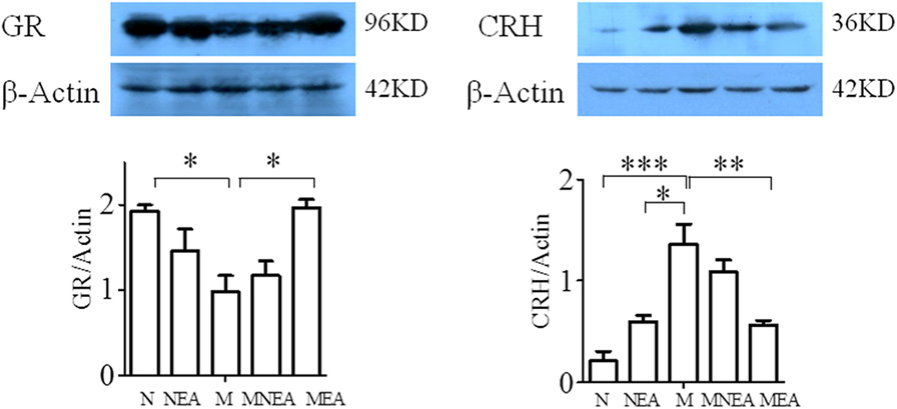
**Effect of acupuncture at the different acupoints on the expressions of GR and CRH protein in PVN.** The vertical ordinate indicated the ratio of GR to β-Actin (the left) and the ratio of CRH to β-Actin (the right). The horizontal ordinate represented the different groups. GR protein expression was obviously reduced in the M group as compare with the N group, while it was dramatically induced in the MEA group as compare with the M group ( #H-300, Santa Cruz, 96KD, dilution: 1:1000). CRH protein expression was obviously increased in the M group as compare with the N and NEA group, while it was obviously reduced in the MEA group as compare with the M group (#10944-1-AP, Datasheet, 36KD, dilution: 1:500).

Experiments compared GR and CRH protein expressions in PVN among the five groups. The results showed: GR protein expression (0.98 ± 0.19) in PVN was significantly reduced and CRH expression (1.36 ± 0.20) was increased apparently in M group, indicating the significant differences as compared with the correspond values in N group(1.93 ± 0.07, 0.21 ± 0.09). Acupuncture promoted GR expression in PVN and the result was different significantly between MEA group and M group (1.97 ± 0.09 *vs* 0.98 ± 0.19, *p* < 0.05). GR expression was also increased in tendency in MNEA group, but without significant difference as compared with M group. GR expression was reduced in tendency in NEA as compared with N group, but without significant difference (Figure 5 left). It was also discovered that CRH protein expression was obviously increased in the M group as compare with the N and NEA group (1.36 ± 0.20 *vs* 0.21 ± 0.09, *p* < 0.001; 1.36 ± 0.20 *vs* 0.6 ± 0.06, *p* < 0.05) separately, while it was obviously reduced in the MEA group as compare with the M group (0.59 ± 0.05 *vs* 1.36 ± 0.20, *p* < 0.01). CRH expression was reduced in tendency in MNEA group, but without significant difference as compared with M group. CRH expression was increased in tendency in NEA group, but there was no significant difference as compared with N group (Figure 5 right).

Taken together, under normal condition, acupuncture could activate PVN to be involved in SRNs to different degrees due to its different effects on distinct acupoints. This activation effect was transient due to the normal function of HPAA. When the stimulation ceased, through HPAA regulation, the activated spikes could be quickly recovered to the spontaneous discharge. This is the reason why we could record different acupoint stimulation effects on the same neuron unit in the first part of experiment. Accordingly, there is no significant differences between N and NEA groups in terms of the behavior, CRH and GR protein expressions in PVN as well as corticosterone level.

Concerning the UCMS rats, HPAA function was of excitatory state, manifested as the increased expression of CRH protein and decreased expression of GR protein in PVN as well as the increased CORT level. Acupuncture inhibited CRH secretion via promoting the expression of GR in PVN so that glucocorticoid level was reduced and HPAA excitation was decreased.

## Discussion

The main achievements of this study: 1) provided a new platform to study the acupoint specificity for acupuncture regulation on HPAA function, 2) induced the decrease of GR expression in PVN under UCMS. Acupuncture up-regulated GR expression in PVN and inhibited CRH secretion so as to reduce CORT level and HPAA excitation.

### PVN participated in the correlation between SRNs and CRH neurons

As an initiator of the endocrine system, the hypothalamus plays a key role in HPAA [20,21]. Neuronal soma of hypothalamic CRH are distributed mainly in PVN. Under stress, the small cell neurons of PVN secrete CRH, and directly activate HPAA [3,22]. Certain amount of glucocorticoids inhibits CRH secretion through feedback inhibition on HPAA. CRH neurons in PVN is the sensitive neurons to the stress stimulation and glucocorticoid.

The study of Song, et al [23] found that paraventricular subnuclei neurons were active during the stress condition, especially the parvocellular subnuclei. In the present experiments, we observed that nearly 25% (163/653) of the neurons recorded in PVN responded to the noxious heat stimulus (48°C), including 42 inhibitory neurons and 121 excitatory ones. The experiments observed the characters of neurons via the feedback regulation of hydrocortisone. The physiological characters of these neurons were similar to those of CRH neurons in PVN [24], indicating their correlation with CRH neurons activities. These neurons are the target neurons in our study.

The acupoints specifically activate SRNs in PVN were also the specificity acupoints for the regulation of HPAA function.

CRH secreted by hypothalamus are the small cell peptidergic neurons. The hypothalamic regulatory peptides are released into the portal system via the projection of the axons to the median eminence. Its axonal endings contact with the first level of capillary in pituitary portal system. These CRH neurons release regulatory peptide into the portal system. Regulating ACTH secretion from pituitary gland and promoting the secretion of CORT in target gland. At the same time, the secretion of CRH neurons in hypothalamus is regulated by the feedback signals and the central nerve system. The regulation of feedback signals includes the short-loop feedback and the long-loop feedback, representing the main type of regulation on CRH secretion. Additionally, CRH itself can produce ultrashort-loop feedback for its self-regulation.

Studies have shown that acupuncture regulated the function of HPAA via promoting hypothalamic CRH secretion so that ACTH and CORT secretion could be regulated [25]. In order to verify the effect of specificity acupoints on regulating HPAA, we examined the behavior, peripheral blood CORT levels, GR and CRH protein expressions in PVN in UCMS model rats under acupuncture at the specificity acupoints and the non-specificity acupoints. The results showed that acupuncture at specificity acupoints modulated the behavior in UCMS rats, down-regulated the level of CORT in peripheral blood, promoted GR expression and suppressed CRH excessive secretion in PVN as compared with M group. It suggests that the acupoints for the specific activation on SRNs in PVN are also the specificity acupoints for the regulation of HPAA function.

## Conclusion

This study provides a new platform to study the specificity of acupoints on acupuncture regulation of HPAA function. Most previous studies using brain slices to explore the effect of different acupoints in vitro, which is difficult to elucidate the relationship between acupoints effect and the dominant spinal cord segments. Since acupuncture leads to an afferent effect, in vivo recording of SRN activation in PVN could objectively describes the effect of acupuncture at different acupoints. Through the effect of specificity acupoint on the behavior, COTR level in peripheral blood, GR and CRH protein expressions in PVN in UCMS mode rat, it is demonstrated in this study that the acupoints specifically activate SRNs in PVN are also the acupoints for the regulation on HPAA function.

In our future study, we will further explore the correlation between acupuncture intervention on HPAA function and the mechanisms regulating GR, CRH or ACTHR.

## Abbreviations

EA: Electro-acupuncture
SRN: Stress reaction neurons
PVN: Hypothalamic paraventricular nucleus
HPAA: Hypothalamic-pituitary-adrenal cortex axis
UCMS: Unpredictable chronic mild stress
